# Changing trends in dialysis modalities utilization and mortality in children, adolescents and young adults with acute kidney injury, 2010–2017

**DOI:** 10.1038/s41598-021-91171-w

**Published:** 2021-06-04

**Authors:** You-Lin Tain, Hsiao-Ching Kuo, Chien-Ning Hsu

**Affiliations:** 1 Department of Pediatrics, Kaohsiung Chang Gung Memorial Hospital, College of Medicine, Chang Gung University, Kaohsiung, 833 Taiwan; 2 Department of Pharmacy, Kaohsiung Chang Gung Memorial Hospital, Kaohsiung, 833 Taiwan; 3 School of Pharmacy, Kaohsiung Medical University, Kaohsiung, 807 Taiwan

**Keywords:** Health care, Nephrology

## Abstract

The aim of the study was to assess trends in the relative use of dialysis modalities in the hospital-based pediatric cohort and to determine risk factors associated with in-hospital morality among pediatric patients receiving dialysis for acute kidney injury (AKI). Patients aged < 20 years who received dialysis between 2010 and 2017 were identified from electronic health records databases of a Taiwan’s healthcare delivery system. The annual uses of intermittent hemodialysis (HD), continuous and automated peritoneal dialysis (PD) and continuous kidney replacement therapy (CKRT) were assessed using Cochran-Armitage Tests for trend. Among patients who received their first dialysis as inpatients for AKI, a multivariate logistic regression model was employed to assess mortality risks associated with dialysis modalities, patient demographics, complexity of baseline chronic disease, and healthcare service use during their hospital stays. Kidney dialysis was performed 37.9 per patient per year over the study period. Intermittent hemodialysis (HD) (73.3%) was the most frequently used dialysis modality. In the inpatient setting, the relative annual use of CKRT increased over the study period, while HD use concomitantly declined (*P* < 0.0001). The overall in-hospital mortality rate after dialysis for AKI was 33.6%, which remained steady over time (*P* = 0.2411). Patients aged < 2 years [adjusted odds ratio: (aOR) 3.36; 95% confidence interval (CI) 1.34–8.93] and greater vasoactive regimen use (aOR: 17.1; 95% CI: 5.3–55.21) were significantly associated with dialysis-related mortality. Overall treatment modality used for dialysis in pediatric patients increased slowly in the study period, and HD and CRKT modality uses largely evolved in the inpatient setting. Younger ages and use of more vasoactive medication regimens were independently associated with increased early mortality in patients on AKI-dialysis.

## Introduction

Acute kidney injury (AKI) is a common complication among hospitalized children worldwide^1–3^. AKI increases the risk of hospital mortality in pediatric patients^3,4^, especially those receiving dialysis^5^ and extracorporeal membrane oxygenation (ECMO) treatments^6^. Dialytic modalities have evolved over time to treat pediatric patients with AKI^7^. These dialytic modalities include continuous ambulatory or automated peritoneal dialysis (PD), intermittent hemodialysis (HD), continuous kidney replacement therapy (CKRT), and hybrid modalities. CKRT has been increasingly used due to its hemodynamic stability in pediatric patients, especially those being treated with HD in intensive care^8^. Although less evidence related with comparative outcomes between CKRT and PD; some evidence demonstrated CKRT is better in the maintenance of fluid balance than PD^7^. However, the trends in the evolving use of various dialytic modalities for treating hospitalized children with AKI have not been well documented.

Previous studies in pediatric patients with AKI receiving dialysis have mainly focused on critical care^5,9^, continuous HD^10,11^, or a specific health condition, such as those requiring cardiac surgery^1,12^; however, these findings may not mirror the changes in overall utilization of dialysis modalities in the pediatric practice. Therefore, we conducted a hospital-based cohort study using an electronic health records database to assess the annual utilization of various dialysis modalities for treating pediatric patients over an eight-year study period. We hypothesized the utilization of kidney dialysis evolved and varied by dialysis modalities over time. The primary aim of the study was to assess utilization rates of three kidney dialysis modalities between 2010 and 2017 among Taiwanese children, adolescents and young adults. The secondary aims were to assess the short-term in-hospital mortality and to understand its relevant risk factors among pediatric patients with AKI receiving dialysis.

## Methods

### Study design and data source

This is a cross-sectional study on dialysis modality utilization in Taiwanese youths under 20 years of age. The study used the Chang Gung Research Database (CGRD), which is an electronic health records database derived from the network of Chang Gung Memorial Hospitals (CGMHs) in Taiwan. CGMHs provide approximately 10–12% of all healthcare services of the Taiwan National Health Insurance (NHI) program^13^. Taiwan’s NHI program is a compulsory, single-payer health insurance program that covers over 99% of Taiwan’s entire population and comprehensive health services^14^. The CGRD contains diagnostic data, prescription history, and laboratory test results from emergency department (ED), inpatient, and outpatient settings, which provides an optimal platform for conducting necessary, ongoing renal research.

The study cohort included patients who received any modality of kidney dialysis at aged < 20 years between January 1, 2010 and December 31, 2017. The study extended 2010–2014 database period in a previous pediatric AKI study to the latest data available for research in the study setting^3^. For some patients with multiple hospitalizations requiring kidney dialysis, only the earliest hospitalization for dialysis-requiring AKI (index hospitalization) was included in in-hospital mortality analysis to improve results interpretation in the inpatient setting.

### Outcomes

The primary outcome measure of the study was the annual utilization rate of three modalities among pediatric patients receiving kidney dialysis therapy in CGMHs. The use of kidney dialysis was defined by at least one billing codes for intermittent HD, PD, or CKRT during the study years by outpatient setting, emergency department (ED), and inpatient setting (Table S1 in the Supplemental file). Some patients transferred from ED to inpatient bed were grouped into the inpatient setting, and for patients discharged from ED were grouped into the outpatient/ED setting. The use of CKRT included continuous venovenous hemodialysis (CVVHD), continuous venovenous hemofiltration (CVVH), and continuous arteriovenous hemofiltration (CAVH).

The secondary outcome measures were short-term in-hospital morality rates and associated risk factors among hospitalized children receiving dialysis for AKI. To avoid AKI misclassification caused by a lack of baseline serum creatinine (SCr) concentration data, International Classification of Diseases and Related Health Problems-9/10 version (ICD-9/10 coded AKI (Table S1 in the Supplemental file) at hospital discharge or SCr-based KDIGO criteria of AKI (peak SCr ≥ 1.5 × admission SCr, or SCr increased to ≥ 4 mg/dL during hospitalization)^15^. An additional secondary outcome measure was the recovery of renal function at the time of hospital discharge defined by a SCr at the date of hospital discharge (or a SCr obtained within 7 days at outpatient visit after the AKI discharge) comparing to the admission SCr as: not recovery (hospital discharge SCr ≥ 1.5 × admission SCr, meeting with stage 1 AKI criterion); recovery (1.2 × to < 1.5 × admission SCr); no change (< 1.2 × admission SCr)^16^. In order to categorize patient characteristics in the mortality analysis, some intermittent HD patients receiving PD therapy were classified into the PD group. Because CKRT only administered in the inpatient setting, patients ever received CKRT and other modalities were classified into the CKRT group.

### Covariates

Potential risk factors of all-cause mortality after dialysis, including patients’ demographic data (sex, age), SCr concentrations, the presence of comorbid conditions, and healthcare services used during the index hospitalization were assessed according to the results of previous studies of pediatric patients receiving dialysis^9,11^. Medical history was assessed based on data from one year prior to the index hospitalization using the Pediatric Medical Complexity Algorithm (PMCA)^17,18^, which can serve as a marker for the complexity of underlying comorbid conditions that can affect in-hospital mortality risk. Prior kidney transplantation was examined by ICD-9th/10th codes (V42.0/Z94.0). Because information about daily doses of a prescriptions used during hospitalization were not available in the CGRD, we summed the number of vasoactive regimen prescribed (i.e., dopamine, dobutamine, norepinephrine, epinephrine, milrinone, and vasopressin); those daily doses were used to calculate a Vasoactive-Inotropic Score (VIS): dopamine dose mcg/kg/min) + dobutamine dose (mcg/kg/min) + 100 × epinephrine dose (mcg/kg/min) + 10 × milrinone dose (mcg/kg/min) + 10,000 × vasopressin dose (units/kg/min) + 100 × norepinephrine dose (mcg/kg/min)^19^ to be used as a proxy measure. Additional known risk factors associated with mortality, ECMO, and intensive care (ICU) use were included in analyses.

### Statistical analysis

The Cochrane Armitage Test for Trend was used to assess trend changes in the relative use of different dialysis modalities over time. All continuous variables are presented as means (standard deviation, SD) and median values (interquartile range [IQR], 25th–75th percentile). Categorical and binary variables are reported as numbers and percentages. Chi-square tests for categorical variables and Analysis of Variance (ANOVA) tests for continuous variables were used to compare baseline data among patients receiving only HD, PD or CKRT therapy. Factors associated with in-hospital mortality were explored from calculating adjusted odds ratios (aORs) with 95% confidence intervals (CI) using multivariate logistic regression modeling based on the covariates mentioned above. To ensure dialysis therapy was considered for AKI during the index hospitalization, patients having had dialysis (including maintenance dialysis for CKD G5) before the index hospitalization were excluded from the sensitivity analysis. All statistical analyses were conducted using SAS (Statistical Analysis System) version 9.4 (SAS Institute, Cary, NC, USA), and all *p* values were two-tailed, with a predetermined alpha level < 0.05 being considered statistically significant.

### Ethics approval

This study proposal was approved by the Institutional Review Boards (IRB) of the Chang Gung Medical Foundation at Taipei, Taiwan (permit number: 201801461B0). The IRB waived the requirement of written informed consent. All methods were performed in accordance with the relevant guidelines and regulations of IRB of Chang Gung Medical Foundation.

## Results

### Primary outcome

#### Use of different kidney dialysis modalities

A total of 23,759 total dialysis encounters of any modality were recorded among the 412 pediatric patients treated from 2010 to 2017 (on average 2970 dialysis encounters per year) with mean 37.87 (± 4.78) encounters per person per year. Intermittent HD accounted for 73.26% of encounters (17,406), followed by 20.15% (4,788 encounters) for PD, and 6.59% (1,565 encounters) for CKRT.

The majority of dialysis encounters were used in ED or outpatient settings (81.91% for 119 patients) and 18% dialysis encounters used for 378 patients in the inpatient setting.

**Figure 1:**
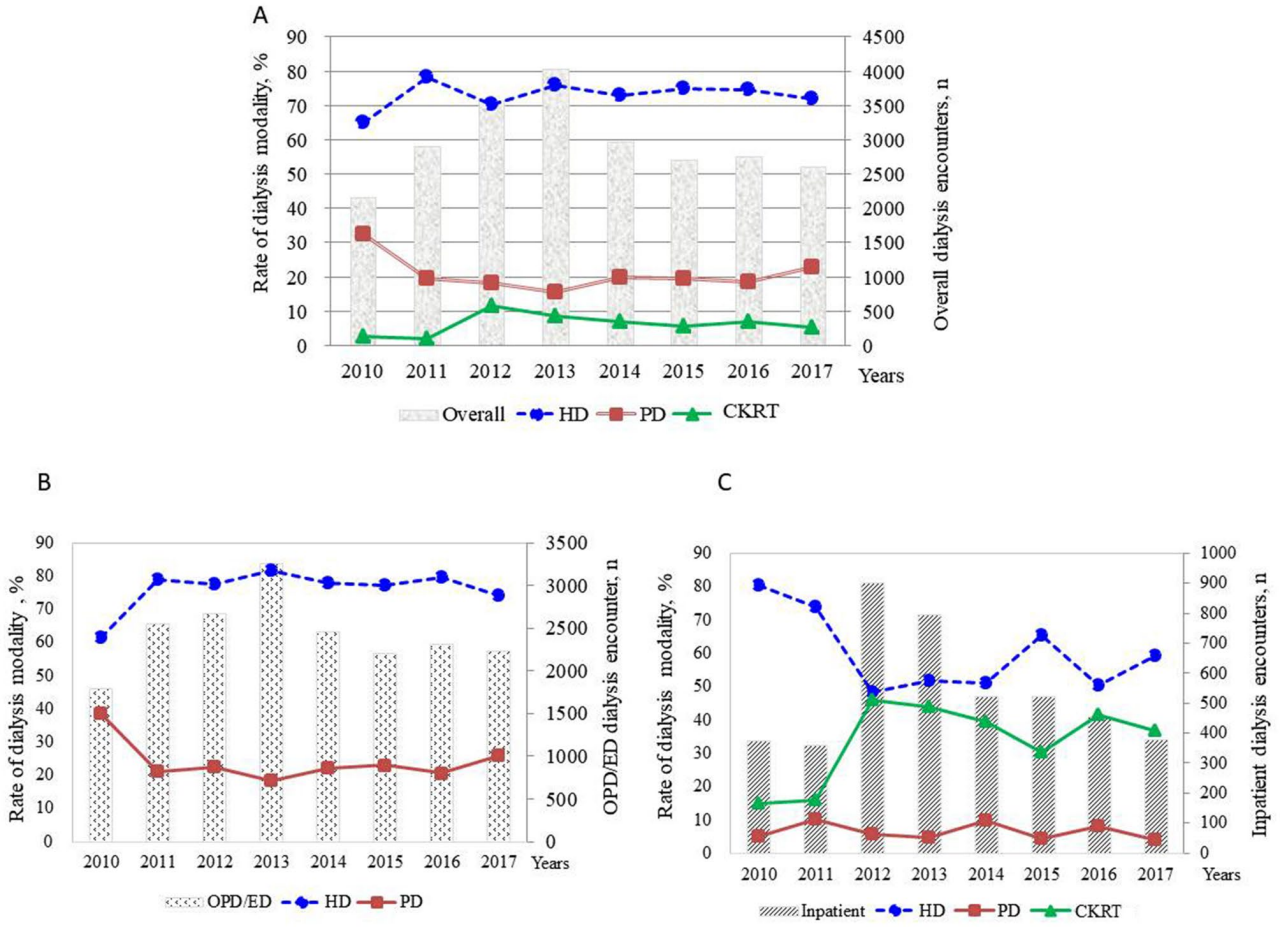
Annual rates of dialysis modality utilization among patients aged < 20 years treated between 2010 and 2017. All data are analyzed by the Cochran-Armitage Test for Trend (Statistical Analysis System version 9.4; SAS Institute, Cary, NC, USA). **(A)** Overall encounters for dialysis (*p* = 0.0204). **(B)** Dialysis encounters in outpatient/emergency department settings (*p* < 0.0001). **(C)** Dialysis encounters in an inpatient setting (*p* < 0.0001).

Figure 1 illustrates the relative use of various modalities per year over the eight-year study period. There was a relative decline in the overall use of dialysis after 2013 in this study’s cohort, as shown in Fig. 1A. When examined as a proportion of relative use across all study years, use of intermittent HD slightly decreased over time, but still accounted for approximately 74% of all dialysis encounters; PD use remained relatively stable and accounted for approximately 20% of all encounters, while CKRT only administered in the inpatient setting and accounted for 5–7% of encounters in more recent years (Cochrane Armitage Test for Trend, P = 0.024; Fig. 1A). In an ED or outpatient setting, the patterns of relative use for intermittent HD and PD were broadly similar; the overall use is shown in Fig. 1B (P < 0.0001). Intermittent HD and CKRT were more frequently employed dialysis modalities in more recent years in an inpatient setting, as shown in Fig. 1C (P < 0.0001).

### Secondary outcome

#### Characteristics of hospitalized patients with AKI-dialysis

Among the 378 patients who received dialysis in an inpatient setting, based on the definition of index hospitalization for dialysis, CKRT was the most frequently employed modality, with 161 (42.60%) patients treated during hospital stays (Table 1), and 21.12% patients who received CKRT (n = 34) were treated by more than one dialysis modality over the study period. Some patients receiving PD were also treated by more than one modality (n = 30, 41.67%). On average, patient age at hospital admission was 10.71 (± 7.4) years. Approximately 40% of dialysis-receiving patients had underlying complex chronic disease (Table 1) and two patients had a history of kidney transplantation.

**Table 1:** Characteristics of hospitalized pediatric patients (n = 378).

	Overall (n = 378)	CKRT (n = 161)	Only HD^#^ (n = 145)	PD (n = 72)	*P* value*
**Sex, n (%)**					0.3821
Boy	209 (55.29)	94 (58.39)	80 (55.17)	35(48.61)	
Girl	169 (44.71)	67 (41.61)	65 (44.83)	37 (51.39)	
**Age at index hospitalization, year, n (%)**					< 0.0001
≤ 1	87 (23.02)	51 (31.68)	3 (2.07)	33 (45.83)	
2 to < 3	17 (4.5)	15 (9.32)	2 (1.38)	0	
3 to < 8	36 (9.52)	24 (14.91)	8 (5.52)	4 (5.56)	
8 to < 13	62 (16.4)	29 (18.01)	24 (16.55)	9 (12.5)	
13 to < 18	118 (31.22)	28 (17.39)	73 (50.34)	17 (23.61)	
18 to < 20	58 (15.34)	14 (8.7)	35 (24.14)	9 (12.5)	
**PMCA, CD condition, n (%) **					0.0483
Without CD	209 (55.29)	101 (62.73)	66 (45.52)	42 (58.33)	
Non-complex CD	18 (4.76)	6 (3.73)	9 (6.21)	3 (4.17)	
Complex CD	151 (39.95)	54 (33.54)	70 (48.28)	27 (37.5)	
**PMCA, organ system, n (%)**					
Cardiac	64 (16.93)	24 (14.91)	27 (18.62)	13 (18.06)	0.661
Dermatological	1 (0.26)	1 (0.62)	0 (0)	0 (0)	0.5088
Endocrinological	12 (3.17)	5 (3.11)	5 (3.45)	2 (2.78)	0.9633
Gastrointestinal	29 (7.67)	15 (9.32)	12 (8.28)	2 (2.78)	0.2097
Genetic	7 (1.85)	2 (1.24)	3 (2.07)	2 (2.78)	0.7024
Genitourinary	14 (3.7)	2 (1.24)	7 (4.83)	5 (6.94)	0.0683
Hematological	39 (10.32)	15 (9.32)	19 (13.1)	5 (6.94)	0.3205
Immunological	30 (7.94)	9 (5.59)	17 (11.72)	4 (5.56)	0.0993
Malignancy	20 (5.29)	16 (9.94)	3 (2.07)	1 (1.39)	0.0023
Mental health	9 (2.38)	4 (2.48)	4 (2.76)	1 (1.39)	0.8182
Metabolic	23 (6.08)	10 (6.21)	9 (6.21)	4 (5.56)	0.9785
Musculoskeletal	10 (2.65)	2 (1.24)	6 (4.14)	2 (2.78)	0.288
Neurological	31 (8.2)	14 (8.7)	13 (8.97)	4 (5.56)	0.659
Ophthalmological	6 (1.59)	2 (1.24)	3 (2.07)	1 (1.39)	0.8369
Otologic	1 (0.26)	0 (0)	1 (0.69)	0	0.4468
Pulmonary/respiratory	15 (3.97)	10 (6.21)	4 (2.76)	1 (1.39)	0.1395
Renal disease	81 (21.43)	10 (6.21)	50 (34.48)	21 (29.17)	< .0001
Progressive^&^	139 (36.77)	45 (27.95)	68 (46.9)	26 (36.11)	0.0028
Prior dialysis, n (%)	22 (9.87)	0	20 (18.35)	2 (5.71)	0.0001

*CKRT* continuous kidney replacement therapy, *HD* hemodialysis, *PD* peritoneal dialysis, *PMCA* pediatric medical complexity algorithm, *CD* chronic disease.

*P value indicates difference among three dialysis modality groups.

^#^Some children received more than one dialysis modalities in the index hospitalization. The CKRT group included some patients receiving CKRT + HD; The PD group included some patients receiving PD + HD.

^$^PMCA and prior dialysis were ascertained among patients with any visits in the study setting 365 days prior to the index hospitalization.

^&^A progressive condition associated with decreased life expectancy (e.g., muscular dystrophy), malignancy, or continuous technology dependence (e.g., dialysis or tracheostomy).

#### Patient outcomes and health services uses

**Figure 2:**
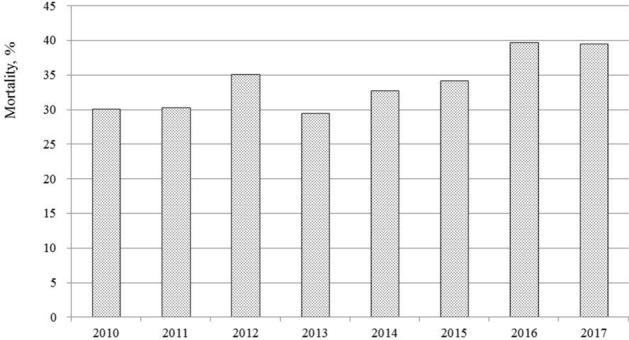
In-hospital mortality after dialysis among patients hospitalized between 2010 and 2017. All data are analyzed by the Cochran-Armitage Test for Trend (p = 0.2411) (Statistical Analysis System version 9.4; SAS Institute, Cary, NC, USA).

Of the hospitalized youths receiving dialysis treatment for AKI, the overall in-hospital mortality rate was 33.6% (n = 127), and increased to nearly 40% (mean 33.80 [± 4.04] per year) (*P* = 0.2411) in 2016–2017 (Fig. 2). Table 2 presents healthcare service use and patient outcomes stratified by dialysis modality. Patients receiving CKRT required more frequent use of ECMO (37.27%), with more patients with the sum of their VIS medication regimens being ≥ 2 (83.85%) compared to other dialysis modalities; this was consistent with needs for ICU care. The median length of stay was 27 days (15–45) among patients receiving dialysis for AKI. The majority of patients (82.47% survivors) had recovery of renal function (discharge SCr 1.2–1.5 × admission SCr) or no change (discharge SCr < 1.2 × admission SCr) at discharge from hospital (Table 2).

**Table 2:** Healthcare services use and in-hospital outcomes.

	Overall (n = 378)	CKRT (n = 161)	Only HD (n = 145)	PD (n = 72)	*P* value*
**Healthcare services use, n (%)**					
ECMO	76 (20.11)	60 (37.27)	5 (3.45)	11 (15.28)	< 0.0001
ICU	302 (79.89)	161 (100)	88 (60.69)	54 (75)	< 0.0001
**VIS medication use**^**#**^					
Dopamine	203 (53.7)	133 (82.61)	35 (24.14)	35 (48.61)	< 0.0001
Dobutamine	98 (25.93)	65 (40.37)	4 (2.76)	29 (40.28)	< 0.0001
(Nor) epinephrine	201 (53.17)	135 (83.85)	30 (20.69)	36 (50)	< 0.0001
Milrinone	119 (31.48)	90 (55.9)	10 (6.9)	19 (26.39)	< 0.0001
**Sum of VIS medication regiment, n (%)**					
≥ 2	191 (50.53)	135 (83.85)	20 (13.79)	36 (50)	<0 .0001
< 2	187 (49.47)	26 (16.15)	125 (86.21)	36 (50)	
**Length of stay, day**					
Mean	40.53 (44.11)	41.48 (43.93)	37.68 (41.17)	44.13 (50.15)	0.5618
Median	27 (15–45)	29 (16–50)	23 (14–39)	31.5 (20.5–46)	
**AKI defined by**^**%**^**, n (%)**					< 0.0001
Increased SCr	288 (76.19)	132 (81.99)	94 (64.83)	62 (86.11)	
Diagnosis codes	70 (18.52)	28(17.39)	35 (24.14	7(9.72	
Diagnosis codes (missing SCr)	20 (5.29)	1 (0.62)	16 (11.03)	3 (4.17)	
**Outcomes, n (%)**					
In-hospital death	127 (33.60)	73 (54.66)	15 (10.34)	24 (33.33)	< 0.0001
Renal function recovery^$^, n	251	134	122	48	
**SCr at hospital discharge, n (%)**					0.0055
Not recovery (≥ 1.5 ×)	23 (9.16)	11 (15.07)	5 (3.84)	7 (14.58)	
Recovery (1.2 × to < 1.5 ×)	18 (7.17)	8 (10.96)	9 (6.92)	1 (2.08)	
No change (< 1.2 ×)	189 (75.30)	53 (72.60)	100 (76.92)	36 (75.00)	
Missing	21 (8.37)	1 (1.37)	4 (8.33)	16 (12.31)	

*CKRT* continuous kidney replacement therapy, *HD* hemodialysis, *PD* peritoneal dialysis, *ECMO* extracorporeal membrane oxygenation, *ICU* intensive care unit, *VIS* vasoactive inotropic score (dopamine dose (mcg/kg/min) + dobutamine dose (mcg/kg/min) + 100 × epinephrine dose (mcg/kg/min) + 10 × milrinone dose (mcg/kg/min) + 10,000 × vasopressin dose (units/kg/min) + 100 × norepinephrine dose (mcg/kg/min), *SCr* serum creatinine level.

*P value indicates difference among three dialysis modality groups.

^#^Number of patients prescribed with vasopressin = 0.

^%^AKI defined by SCr-based criteria during hospital stay or diagnosis codes at hospital discharge with and without (missing) SCr data.

^$^Renal function recovery in survivors based on discharge/admission SCr: ≥ 1.5 (no recovery), 1.2- 1.5 (recovery), and < 1.2 (no change).

#### Risk factors associated with in-hospital mortality

In-hospital mortality was significantly associated with a patient age less than two years (aOR 3.46, 95%CI, 1.34–8.93, *P* = 0.0105) and VIH medication use, including one or two medications (aOR 10.8, 95%CI,3.51–33.20) and three to four medications (aOR 17.10, 95% CI,5.30–55.21). Dialysis modality was not significantly associated with mortality, although patients receiving CKRT had a higher risk of mortality than those treated by PD or HD alone (aOR 2.16 [95% CI 0.98–4.76] vs aOR 1.84 [95%CI 0.63–5.38], respectively) (Table 3). On the other hand, patients with eGFR < 15 ml/min/1.73m^2^ (aOR 0.33, 95%CI, 0.12–0.91) and missing SCr (aOR 0.64, 95%CI, 0.18–2.29) at the admission were less likely associated with in-hospital mortality (Table 3). The significant mortality risks remained similar in the sensitivity analyses when patients were excluded for having received prior dialysis therapy (n = 22) (Table S2 in the Supplemental file).

**Table 3:** Factors associated with mortality among patients receiving dialysis for acute kidney disease (n = 378).

	Deceased (n = 127)	Survivor (n = 251)	aOR (95%CI) P value		
**Sex, n (%)**					
Boy	72 (56.69)	137 (54.58)	1		
Girl	55 (43.31)	114 (45.42)	0.84 (0.49, 1.46)		0.5376
**Age at index hospitalization, year, n (%)**					
≥ 13	39 (30.71)	154 (61.35)	1		
2–12	34 (26.77)	64 (25.50)	1.62	(0.68, 3.85)	0.2735
< 2	54 (42.52)	33 (13.15)	3.46	(1.34, 8.93)	0.0105
**ECMO, n (%)**					
No	79 (62.20)	223 (88.84)	1		
Yes	48 (37.80)	28 (11.16)	1.28 (0.68, 2.41)		0.4526
**AKI defined by, n (%)**^**#**^					
Diagnosis codes	20 (15.75)	50 (19.92)	1		
Increased SCr	107 (84.25)	181 (72.11)	1.60 (0.77, 3.35)		0.2077
Diagnosis codes (missing SCr)	0	20 (0.80)			
**Sum of VIS medication regiment, n (%)**					
None	4 (0.32)	138 (54.98)	1		
1–2	40 (31.50)	64 (25.50)	10.80 (3.51, 33.20)		< .0001
3–4	83 (65.35)	49 (19.52)	17.10 (5.30, 55.21)		< .0001
**Dialysis modality, n (%)**					
Only HD	15 (11.81)	130 (51.79)	1		
PD	88 (69.29)	73 (29.08)	1.84 (0.63, 5.38)		0.2644
CKRT	24 (18.90)	48 (19.12)	2.16 (0.98, 4.76)		0.0557
**eGFR at admission, ml/min/1.73 m**^**2**^**, n (%)**					
≥ 60	18 (14.17)	34 (13.55)	1		
15–60	8 (0.06)	18 (0.07)	1.02 (0.29, 3.56)		0.9772
< 15	78 (61.42)	168 (66.93)	0.33 (0.12, 0.91)		0.0323
Missing	23 (18.11)	31 (12.35)	0.64 (0.18, 2.29)		0.4892
**PMCA, organ system, n (%)**					
**Renal disease**					
No	120 (94.49)	177 (70.52)	1		
Yes	7 (0.06)	74 (29.48)	0.37 (0.12, 1.13)		0.0799
**Progressive**^**&**^					
No	90 (70.87)	149 (59.36)	1		
Yes	37 (29.13)	102 (40.64)	1.53 (0.75, 3.11)		0.2394

*aOR* adjusted odds ratio, *EMCO* extracorporeal membrane oxygenation, *SCr* serum creatinine level, *VIS* vasoactive inotropic score (dopamine dose (mcg/kg/min) + dobutamine dose (mcg/kg/min) + 100 × epinephrine dose (mcg/kg/min) + 10 × milrinone dose (mcg/kg/min) + 10,000 × vasopressin dose (units/kg/min) + 100 × norepinephrine dose (mcg/kg/min), *eGFR* estimated glomerular filtration rate, *PMCA* pediatric medical complexity algorithm, *CD* chronic disease.

^#^AKI defined by SCr-based criteria during hospital stay or diagnosis codes at hospital discharge with and without (missing) SCr data.

^&^A progressive condition associated with decreased life expectancy (e.g., muscular dystrophy), malignancy, or continuous technology dependence (e.g., dialysis or tracheostomy).

## Discussion

This hospital-based cohort study demonstrated that the overall utilization of kidney dialysis in pediatric patients increased and the trends in the relative use of intermittent HD, PD and CKRT changed over time, which was mostly likely due to increased use of CKRT in an inpatient setting. The study results suggest that patient aged younger than 2 years and uses of VIS regiment were associated with increased early mortality risk and may be important to improve dialysis outcomes. Approximately 9.2% of AKI patients had non-recovery kidney function at hospital discharge after dialysis therapy, highlights the importance of post-AKI care in pediatric practice.

A previous study reported the incidence of dialysis in pediatric patients treated for AKI increased by 7% per year (2000–2009) in the pediatric population of the United States using ICD-9 coded AKI combined procedure codes for dialysis^20^. The incidence of dialysis-requiring AKI was defined by acute procedure coder for dialysis used in the health care system and found to slightly decline from 0.03/100 person-years to 0.02/100 person-years (1996–2015) in a Canadian pediatric population in the province of Ontario^21^. Although cases of pediatric patients with dialysis for AKI slightly increased (Fig. 1C) over the study period (dialysis encounters from 340 in 2010 increased to 374 in 2017) in the present study with more recent data, it cannot be interpreted as an incidence of dialysis-requiring AKI as not all hospitalized patients in the specific calendar year were included in denominator.

It is worth mentioning that different definitions of AKI would affect the number of AKI cases and utilization rate of dialysis-requiring AKI. Several studies have showed inadequate validity and accuracy of using ICD discharge coding to identify AKI^22–25^. Despite SCr-based defined AKI is a relatively new concept to allow early identification and prevention of patients at risk for AKI, we found ICD-9 discharge coding for AKI were sensitive to hospitalized patients with more severe stage of SCr-based defined AKI^23^. For dialysis-requiring AKI cases in pediatric patients, we found only 5.3% patients with insufficient SCr data to define AKI. Additionally, ICD procedure coding (ICD-9 39.95 or ICD-10 Z99.2) cannot discriminate intermittent or continuous dialysis modality, we used Taiwan NHI billing codes and further found the utilization rate of CKRT increased over time in pediatric practice.

Among hospitalized patients who underwent dialysis, the sex and age distribution of this study’s cohort are similar to previous studies from single-center^9^ and population-based cohorts^21^. However, the patterns of dialysis modality utilization appeared to change by the time CKRT became more accessible in different countries. For instance, a recent survey of 35 pediatric nephrology centers in 11 European countries reported that PD and CKRT were the most frequently used dialysis modalities for treating pediatric AKI, accounting for 39.4% and 38.2% of all treatments, followed by use of intermittent HD (22.4%)^26^. Over the period of 2010–2015, CKRT (with an annual rate ranging from 38 to 52%) and intermittent HD (annual rate ranging from 12 to 24%) were more commonly performed in pediatric patients in Ontario^21^. Our results showed that intermittent and CKRT were the predominant dialysis modalities for hospitalized pediatric patients. We also found that AKI patients receiving CKRT were younger than those treated by other dialytic modalities. This trend is presumably due to significant advances in CKRT technology applied in pediatric populations. As new marketed CKRT machines require smaller extracorporeal volumes, it is expected that CKRT will become more accessible for critically ill infants in the future^27^. It is noticed that the peak of CKRT utilization in 2012 inpatient setting might be related to the nationwide enterovirus 71 (EV71) epidemics occurred in that year^28,29^. CKRT was a rescue therapy for critically ill infants and children with EV71 infection and the marked increase in continuous HD utilization declined after 2012 as the EV71 infected cases were controlled.

Our results are consistent with previous studies that reported the overall mortality in pediatric patients with dialysis for AKI ranged from 19 to 44.7%^5,8,9,21,30^. Results from a present study demonstrated that patients receiving CKRT required more frequent use of ECMO and more vasoactive inotropic medications (based on the sum of VIS medication regimens being ≥ 2), which is consistent with needs for ICU care as well as higher mortality risk. The associations between inotropic mediation use and mortality in cases of AKI have been documented in patients mainly from small, single-center studies^9,11^, but this was not reported by some other studies, such as those in which neonates underwent cardiac surgery or in those with multi-organ dysfunction syndrome^12,31^. In addition, medical complexity accounts for a large proportion of hospital admissions, ICU stays, and increased mortality rates^32,33^. Using PMCA, our study is the first to evaluate whether the different level of complexity of comorbidities or organ system involved is associated with mortality in patients receiving dialysis; we demonstrated that nearly 40% of pediatric patients had complex chronic disease and progressive condition (36.77%) including 8 patients with organ transplantation history (2 with kidney transplantation). Patients with eGFR < 15 ml/min/1.73 m^2^ at admission might be more severe stage of AKI at admission and was treated with urgent intensive interventions resulting to a lower in-hospital mortality risk. Because the choice of initial dialysis modality was not assessed in the present study, the odds between a variety of dialysis modality and early mortality should be interpreted with caution. Currently, it is unclear what patients may benefit from the PD rather than HD or CRRT in specific circumstances. Additional studies are warranted to identify risk factors for all-cause mortality and to validate treatment modality administration protocols in patients with AKI requiring dialysis.

The present study had several strengths: First, all data were collected from an electronic health records database in a general sample of pediatric patients with dialysis therapy. These data demonstrate the changing patterns of dialysis modality utilization in a routine pediatric practice setting in a Asia population. Second, we used PMCA to differentiate the complexity of comorbid conditions in patients receiving dialysis and evaluated its effects on mortality outcomes. In addition to VIS medication use, these results suggested that assessing data *prior* to dialysis treatment and during ICU care for AKI are important and should be taken into accounted in all dialysis outcome research. Time to kidney recovery and the extent of recovery following AKI have been suggested to be associated with adverse long-term outcomes^34,35^. Although 54.8% survivors (n = 207) met less than 1.5 × peak SCr criterion (stage 1 AKI criterion), most of them (n = 189) were with < 1.2 × peak SCr criterion. Importantly, 9.2% patients were non-recovery and 8.4% patients without follow-up SCr data might be less likely to have full recovery of kidney function. Despite the non-recovery criterion is the first used to examine early recovery in pediatric patients with dialysis-requiring AKI, these study results carry important implications in prognosis and post-AKI care in pediatric population. In considering further research focusing patients who still require dialysis after hospital discharge, length of time continued on dialysis and incidence and risk factors for transition from AKI to CKD can help determine when is critical to promote continuation of post-AKI care (e.g. 90 days)^36^ to improve long-term patient outcomes.

Our study had several limitations. First, the electronic health records database used was derived from a single healthcare delivery system, and the dialysis outcomes may not be generalizable to other practice settings. Second, the nature of our electronic health records data did not allow us to account for some reportedly important factors due to either a lack of data availability or the fact that some data that are not collected as part of routine care practice. For instance, it is noteworthy that most ICU care data are not currently structurally recorded and may be unavailable for analysis in electronic health records databases for hospitalized patients experiencing AKI. Whether dialysis was applied for toxins/drugs removal and the causes of dialysis-requiring AKI were not evaluated in the present study, so future research is undoubtedly warranted.

In conclusion, the present study demonstrated that overall utilization of kidney dialysis increased 21% between 2010 and 2017. Intermittent HD and CKRT were major dialysis modalities, with an increasing trend in relative CKRT use in hospitalized pediatric patients treated for AKI. Among pediatric patients with AKI requiring dialysis, age, progression of chronic disease, ICU care, ECMO, and vasoactive inotropic agents use should be considered in the development of adequate criteria for selecting dialysis modalities; a standardized treatment protocol would help optimize therapy selection and improve dialysis outcomes.

## Supplementary Information

Supplementary Tables.
