# Supplementary material for: Changing trends in dialysis modalities utilization and mortality in children, adolescents and young adults with acute kidney injury, 2010–2017
Source: Sci Rep. 2021 Jun 4;11:11887. doi: 10.1038/s41598-021-91171-w (PMC8178371; doi:10.1038/s41598-021-91171-w)
Supplement: Supplementary file 1 — Supplementary Tables. [file 41598_2021_91171_MOESM1_ESM.docx]

**Changing Trends in Dialysis Modalities Utilization and Mortality in Children, Adolescents and Young Adults with Acute Kidney Injury, 2010-2017**

You-Lin Tain, Hsiao-Ching Kuo, Chien-Ning Hsu

**Supplemental file**

Table S1. Codes and definitions of study covariates

Table S2. Sensitivity analysis: Factors associated with mortality among hospitalized patients receiving dialysis for acute kidney injury (n=356)

Table S1. Codes and definitions of study covariates

| Disease/healthcare service | Codes | Definition |
| --- | --- | --- |
| Dialysis | Billing codes for dialysis based on Taiwan National Health Insurance program  HD: 58001C, 58003C, 58027C, 58029C,58030B  PD: 58002C, 58009B, 58011C, 58017C, 58028C.  CKRT: 58007C, 58014C, 58018C | Encounters for dialysis in outpatient, emergency department and inpatient settings between 2010 and 2017. Intermittent hemodialysis (HD)  Peritoneal dialysis (PD)  Continuous kidney replacement therapy (CKRT): venovenous hemodialysis (CVVHD), continuous venovenous hemofiltration (CVVH), and continuous arteriovenous hemofiltration (CAVH) |
| Acute kidney injury (AKI) | ICD-9-CM: 584.9 (2010-2016)  ICD-10-CM: N17.9 (2017) | Diagnosis at discharge from hospital |
|  | SCr value | Peak SCr during hospitalization≧1.5× admission SCr, or SCr≧4mg/dL |
| Extracorporeal membrane oxygenation (ECMO) | ICD-9-CM procedure: 39.65 or billing codes used in the study setting | During hospitalization |
| Pediatric Medical Complexity Algorithm (PMCA) | ICD9/10-CM | ICD codes for disease diagnosis in outpatient or inpatient setting, 1-year prior to the index hospitalization |
| Vasoactive inotropic score (VIS) medication by ATC codes |  | (Dopamine dose (mcg/kg/min) + Dobutamine dose (mcg/kg/min) + 100 x Epinephrine dose (mcg/kg/min+ 10 x Milrinone dose (mcg/kg/min) + 10,000 x Vasopressin dose (units/kg/min) + 100 x Norepinephrine dose (mcg/kg/min) |
| Norepinephrine | C01CA03 | During hospitalization |
| Dopamine | C01CA04 | During hospitalization |
| Dobutamine | C01CA07 | During hospitalization |
| Epinephrine | C01CA24 | During hospitalization |
| Milrinone | C01CE02 | During hospitalization |
| Vasopressin | H01BA01 | During hospitalization |
| Renal function recovery |  | SCr change at between the date of hospital discharge (or +7 days post-discharge) and the date at hospital admission:  Not recovery (≥1.5×) ; Recovery (1.2× to <1.5×); No change (<1.2×) |

Abbreviations: ICD-9/10-CM: International Classiﬁcation of Diseases, Ninth and Tenth Revisions, Clinical Modiﬁcation; SCr, serum creatinine level; ATC: anatomical therapeutic chemical classification system.

Table S2 Sensitivity analysis: Factors associated with mortality among hospitalized patients receiving dialysis for acute kidney injury (n=356)

|  |  | | Deceased  (n=127) | |  | Survivor (n=229) | | aOR (95%CI)  *P value* | | | | |
| --- | --- | --- | --- | --- | --- | --- | --- | --- | --- | --- | --- | --- |
| Sex, n (%) | | |  |  |  |  |  |  |  |  |  |  |
|  | Boys | | 72 | (56.69) |  | 125 | (54.59) | 1 | | | |  |
|  | Girls | | 55 | (43.31) |  | 104 | (45.41) | 0.85 | (0.49 |  | 1.46) | 0.5476 |
| Age at index hospitalization, year, n (%) | | | | |  |  |  |  |  |  |  |  |
|  | ≥13 | | 39 | (30.71) |  | 136 | (59.39) | 1 | | | |  |
|  | 2-12 | | 34 | (26.77) |  | 60 | (26.20) | 1.61 | (0.68 |  | 3.82) | 0.2841 |
|  | <2 | | 54 | (42.52) |  | 33 | (14.41) | 3.46 | (1.34 |  | 8.96) | 0.0104 |
| ECMO, n (%) | | |  |  |  |  |  |  |  |  |  |  |
|  | No | | 79 | (62.20) |  | 201 | (87.77) | 1 | | | |  |
|  | Yes | | 48 | (37.80) |  | 28 | (12.23) | 1.28 | (0.68 |  | 2.41) | 0.4472 |
| AKI defined by, n(%)^#^ | | | | | | |  |  |  |  |  |  |
|  | Diagnosis codes | | 20 | (15.75) |  | 50 | (21.83) | 1 | | | |  |
|  | Increased SCr | | 107 | (84.25) |  | 162 | (70.74) | 1.62 | (0.78 |  | 3.38) | 0.1961 |
|  | Diagnosis codes (missing SCr) | | 0 |  |  | 17 | (7.42) |  |  |  |  |  |
| Sum of VIS medication regiment, n (%) | | | | |  |  |  |  |  |  |  |  |
|  | None | | 4 | (3.15) |  | 120 | (52.40) | 1 | | | |  |
|  | 1-2 | | 40 | (31.50) |  | 60 | (26.20) | 10.60 | (3.44 |  | 32.66) | <.0001 |
|  | 3-4 | | 83 | (65.35) |  | 49 | (21.40) | 16.64 | (5.15 |  | 53.75) | <.0001 |
| Dialysis modality, n (%) | | | | |  |  |  |  | | | |  |
|  | Only HD | | 15 | (11.81) |  | 110 | (48.03) | 1 | | | |  |
|  | PD | | 88 | (69.29) |  | 73 | (31.88) | 1.72 | (0.58 |  | 5.04) | 0.3272 |
|  | CKRT | | 24 | (18.90) |  | 46 | (20.09) | 2.05 | (0.93 |  | 4.53) | 0.0754 |
| eGFR at admission, ml/min/1.73m^2^, n (%) | | | | |  |  |  |  |  |  |  |  |
|  | ≥60 | | 18 | (14.17) |  | 34 | (14.85) | 1 | | | |  |
|  | 15-60 | | 8 | (6.30) |  | 18 | (7.86) | 1.01 | (0.29 |  | 3.48) | 0.9925 |
|  | <15 | | 78 | (61.42) |  | 149 | (65.07) | 0.34 | (0.12 |  | 0.95) | 0.0385 |
|  | Data missing | | 23 | (18.11) |  | 28 | (12.23) | 0.66 | (0.18 |  | 2.36) | 0.5193 |
| PMCA, organ system, n (%) | | | | |  |  |  |  |  |  |  |  |
|  | Renal disease | |  |  |  |  |  |  |  |  |  |  |
|  |  | No | 120 | (94.49) |  | 175 | (74.62) | 1 | | | |  |
|  |  | Yes | 7 | (5.51) |  | 54 | (23.58) | 0.42 | (0.14 |  | 1.29) | 0.1304 |
|  | Progressive^&^ | |  |  |  |  |  |  |  |  |  |  |
|  |  | No | 90 | (70.87) |  | 147 | (64.19) | 1 | | | |  |
|  |  | Yes | 37 | (29.13) |  | 82 | (35.81) | 1.53 | (0.75 |  | 3.10) | 0.2397 |

Abbreviations: aOR: adjusted odds ratio; EMCO, extracorporeal membrane oxygenation; SCr, serum creatinine level; VIS, vasoactive inotropic score (dopamine dose (mcg/kg/min) + dobutamine dose (mcg/kg/min) + 100 x epinephrine dose (mcg/kg/min) + 10 x milrinone dose (mcg/kg/min) + 10,000 x vasopressin dose (units/kg/min) + 100 x norepinephrine dose (mcg/kg/min); eGFR, estimated glomerular filtration rate; PMCA, Pediatric Medical Complexity Algorithm; CD, chronic disease; eGFR, estimated glomerular filtration rate; PMCA, Pediatric Medical Complexity Algorithm; CD, chronic disease.

# AKI defined by SCr-based criteria during hospital stay or diagnosis codes at hospital discharge with and without (missing) SCr data

& a progressive condition associated with decreased life expectancy (e.g., muscular dystrophy), malignancy, or continuous technology dependence (e.g., dialysis or tracheostomy).
